# Circulating C-terminal peptides and polymers of alpha-1 antitrypsin as putative markers of pediatric Pi*ZZ liver disease

**DOI:** 10.3389/fped.2025.1717317

**Published:** 2025-12-10

**Authors:** David Katzer, Friedemann Robert Börner, Alexander Weigert, Julia Held, Soyhan Bagci, Rainer Ganschow, Pia Mielczarek, Alexander Machui, Michael Kiehntopf, Sabina Janciauskiene

**Affiliations:** 1 Department of Pediatric Gastroenterology and Hepatology, University Hospital of Bonn Children’s Hospital, Bonn, Germany; 2 Institute of Clinical Chemistry and Laboratory Diagnostics, Jena University Hospital, Jena, Germany; 3 Department of Respiratory and Infectious Diseases, Member of the German Center for Lung Research (DZL), Biomedical Research in Endstage and Obstructive Lung Disease Hannover (BREATH), Hannover Medical School, Hannover, Germany; 4 Department of Neonatology and Pediatric Intensive Care Medicine, University Hospital of Bonn Children’s Hospital, Bonn, Germany; 5 Institute for Digital Medicine, University Hospital Bonn, Bonn, Germany; 6 Institute for Medical Biometry, Informatics and Epidemiology, University Hospital Bonn, Bonn, Germany

**Keywords:** alpha-1-antitrypsin deficiency, pediatric liver disease, biomarkers, AAT peptides, AAT polymers, liquid chromatography–tandem mass spectrometry

## Abstract

**Objective:**

Severe Pi*ZZ alpha-1 antitrypsin (AAT) deficiency, caused by the Glu342Lys mutation in the *SERPINA1* gene, resulting in protein misfolding and polymerization in hepatocytes, and proteotoxic stress which may lead to progressive liver injury. Although liver disease can appear in both childhood and adulthood, most children remain asymptomatic, but no reliable circulating biomarkers currently predict disease progression. In this exploratory study, we aim to assess C-terminal AAT peptides and AAT polymers as potential plasma markers of liver status in clinically stable Pi*ZZ children.

**Methods:**

Plasma from 20 Pi*ZZ children was analyzed by liquid chromatography–tandem mass spectrometry for nine C-terminal AAT peptides and by western blot for AAT polymers. Associations with liver enzymes, ursodeoxycholic acid use, age, and clinical severity scores were evaluated.

**Results:**

Peptides C36, C37, and C42 were consistently detectable in plasma. C37 and C42 levels negatively correlated with age and positively with alanine aminotransferase; C42 additionally correlated with aspartate aminotransferase. Both C37 and C42 were reduced in children receiving ursodeoxycholic acid, whereas C36 showed no significant associations. Circulating AAT polymers correlated with *γ*-glutamyl transferase but not with alanine or aspartate aminotransferase. No correlations were observed between peptide and polymer levels.

**Conclusion:**

Peptides C37 and C42 emerge as promising AAT-derived markers of hepatocellular status in stable Pi*ZZ children, while AAT polymers appear to reflect distinct features of cholestatic or hepatocellular stress. Together, these markers may support early risk stratification and therapeutic monitoring in pediatric AATD-related liver disease. Validation in larger, longitudinal cohorts is warranted to confirm their clinical utility.

## 1 Introduction

Alpha-1 antitrypsin deficiency (AATD) is an autosomal codominant inherited condition caused by mutations in the *SERPINA1* gene. More than 500 distinct *SERPINA1* variants have been identified to date, although only a subset is implicated in clinical disease (1). Among these, the Pi*ZZ genotype is the most clinically relevant, affecting approximately 1 in 2,000 individuals of European descent (2). This genotype results from homozygosity for the Z (Glu342Lys) allele, which leads to the misfolding and polymerization of the alpha-1 antitrypsin (AAT) protein. The misfolded AAT accumulates in the endoplasmic reticulum of hepatocytes, causing proteotoxic stress and progressive liver damage—a gain-of-function effect (3). At the same time, the secretion of functional AAT into the bloodstream is markedly reduced, impairing its protective role against neutrophil proteases in the lungs. As a result, individuals with AATD are predisposed to developing pulmonary emphysema from an early age, particularly when additional adverse cofactors, such as smoking, environmental exposures or unhealthy lifestyle habits, are present (2, 4).

Liver disease associated with the Pi*ZZ genotype shows a bimodal age distribution: it often presents in early childhood, frequently as neonatal cholestasis shortly after birth, and reappears in later adulthood (5). The reasons why most adolescents and young adults remain free of significant liver disease are not well understood. Similarly, the factors that determine the highly variable course of liver disease in childhood remain unclear. Fewer than 5% of affected children develop severe liver disease and require liver transplantation (6).

With several promising therapies for AATD-related liver disease now in development including RNA interference–based silencing of mutant *SERPINA1*, gene replacement strategies, and hepatocyte-directed proteostasis modulators, identifying reliable prognostic factors has become increasingly important to define which patients are most likely to benefit and to optimize clinical trial design (7, 8). To date, clinical research has predominantly focused on adult populations. Early identification of pediatric patients at risk of a severe liver disease course would be of considerable value, as it would facilitate their inclusion in clinical trials and enable timely therapeutic intervention. Some studies have suggested that neonatal cholestasis is more common in patients who later experience a poor clinical course (9); however, this association has limited sensitivity and specificity, since many infants with cholestasis recover without progressive disease, while others without early cholestasis may still go on to develop significant liver injury.

A very small proportion (<1%) of the AAT polymers formed in hepatocytes can also be found in the bloodstream of Pi*ZZ carriers and circulating polymer levels have been shown to correlate with the severity of lung and liver disease in adults (10–14). Elevated polymer levels have been linked to an increased risk of adverse clinical outcomes of liver disease in adult and pediatric patients with AATD (12, 15).

Although the Pi*ZZ mutation reduces plasma levels of AAT, the reactive center loop, which is shared with the wild-type AAT genotype (Pi*MM) and contains the protease binding site, is relatively preserved, allowing Z-AAT to retain the ability to inhibit neutrophil proteases (16). As a result, proteolytic cleavage of wild type M- or Z-AAT proteins results in the formation of larger N-terminal fragments and short C-terminal peptides. AAT peptides represent potential diagnostic biomarkers in sepsis and COVID-19, and plasma peptide levels could be used to predict mortality in critically ill patients (17–20). In addition, AAT-derived peptides exhibit distinct biological activities. The peptide corresponding to the last 42 amino acids of AAT named C42, is capable of inducing hepatocellular dysfunction in a liver-on-chip model, while peptide C36 influences bile acid synthesis in mice (18, 21).

In adult patients with Pi*ZZ-related lung disease, we demonstrated that circulating AAT peptide levels are closely linked to plasma AAT concentrations, with markedly reduced levels observed in individuals with AATD who were not receiving augmentation therapy (22). However, pediatric liver disease in AATD arises independently of lung manifestations, and the levels of AAT-derived peptides in Pi*ZZ-related liver disease have not yet been defined.

To identify AAT-related markers of liver injury or disease progression, we analyzed circulating AAT polymers and C-terminal AAT peptides in children with clinically stable Pi*ZZ AATD. Our aim was to assess whether these markers correlate with established clinical indicators of liver disease severity and could therefore serve as prognostic tools in pediatric AATD.

## 2 Material and methods

### 2.1 Study design and participants

This retrospective cross-sectional study analyzed EDTA-plasma samples from 20 children with confirmed severe AATD (Pi*ZZ), obtained from our institutional biobank. Sample selection was based on documented genotyping and a low AAT concentration in blood. Samples were collected during routine follow-up visits at the pediatric outpatient clinic from June 2022 to July 2024. At the same time, standard clinical assessments were conducted, including liver function tests and a broad panel of hematological and biochemical parameters, including liver markers (Table 1). In addition, a detailed medical history was obtained. Patients receiving ursodeoxycholic acid (UDCA) were generally treated with doses of approximately 250–500 mg per day, corresponding to 10–15 mg/kg body weight.

**Table 1:** Patient and clinical characteristics.

Criteria	*N*=	Mean (SD)	Median (IQR)	Min, Max
Age [years]	20	7.24 (4.27)	8.33 (6.96)	0.500, 14.4
ALT [U/L]	20	61.4 (38.0)	55.5 (30.0)	14.0, 193
AST [U/L]	20	57.3 (26.7)	53.0 (25.0)	32.0, 147
GGT [U/L]	19	21.4 (10.8)	17.0 (9.50)	11.0, 45.0
Bile acid [μmol/L]	13	14.3 (8.14)	13.4 (11.0)	4.30, 31.2
Thrombocytes [G/L]	20	319 (78.9)	300 (77.0)	182, 505
Sex				
Male (%)	11 (55%)			
Female(%)	9 (45%)			
UDCA therapy				
No (%)	8 (40%)			
Yes (%)	12 (60%)			
Icterus prolongatus				
No (%)	12 (60%)			
Yes (%)	8 (40%)			
Total AAT (categorized)				
≥0.3 g/L (%)	13 (65%)			
<0.3 g/L (%)	6 (30%)			
Missing (%)	1 (5.0%)			

AAT, alpha-1-antitrypsin; ALT, alanine aminotransferase; AST, aspartate aminotransferase; GGT, γ-glutamyltransferase; IQR, interquartile range; Max, maximum; Min, minimum; SD, standard deviation; UDCA, ursodeoxycholic acid.

The classification of liver disease stages in this study followed the system established in our previous publication (23). Patients were assigned to different categories based on their liver status at the time of sample collection. In this cohort, only individuals with mild or moderate liver disease without portal hypertension were included. The detailed classification of liver disease stages is provided in the Supplementary Table S1.

### 2.2 Measurement of total AAT in patient plasma

Plasma AAT concentrations were determined using the tina-quant α1-Antitrypsin ver.2 assay (Roche Diagnostics GmbH, Mannheim, Germany), an immunoturbidimetric test performed on cobas c systems. The assay has a measuring range of 0.2–6.0 g/L with a limit of quantification of 0.3 g/L, and calibration is traceable to the certified reference material ERM-DA470k/IFCC.

### 2.3 Analysis of Z-AAT polymers by Western Blot

The molecular forms of purified Pi*ZZ AAT and AAT present in patient plasma were analyzed by native PAGE followed by Western blotting. Plasma samples were diluted 1:10 in phosphate-buffered saline (PBS, pH 7.2) and mixed 1:1 (v/v) with 2× sample buffer lacking detergents. Samples were separated on 7.5% native polyacrylamide gels (40% Acrylamide/Bis Solution 29:1, Bio-Rad Laboratories Inc., Hercules, CA, USA) and subsequently transferred onto polyvinylidene fluoride (PVDF, Merck Millipore, Merck KGaA, Darmstadt) membranes. Polymeric Z-AAT was detected using the monoclonal anti-Z-AAT polymer antibody LG96 (12.5 µg/mL; Candor Biosciences; deposited under accession number DSM ACC3092 at the German Collection of Microorganisms and Cell Cultures), followed by incubation with horseradish peroxidase–conjugated polyclonal rabbit anti-mouse immunoglobulins (0.2 µg/mL; DAKO, Glostrup, Denmark). Immune complexes were visualized using the Clarity Western ECL substrate (Bio-Rad) and imaged with the ChemiDoc Touch imaging system. For semi-quantitative assessment of polymer levels, band intensities were analyzed using Image Lab 6.1 software (Bio-Rad). To ensure reproducibility and standardization across experiments, each gel contained an identically processed Z-AAT control sample with a known amount of polymerized AAT (1.5 µg). Polymer concentrations in patient plasma (mg/dL) were calculated by comparing sample signal intensities to this internal standard run in parallel on the same Western blot.

### 2.4 Analysis of AAT peptides

For the analysis of AAT peptides by liquid chromatography—tandem mass spectrometry (LC-MS/MS), 35 µL of patient EDTA plasma were mixed with 10 µL of internal standard mixture (isotopic labelled peptides C22, C37 and C42; 0,8 µM each, sb-PEPTIDE, Saint Egréve, France) and prepared as stated elsewhere (17). Calibration standards and quality control samples of synthetic peptides were processed accordingly. The stepwise gradient for chromatographic separation on a Shimadzu ultra-high performance liquid chromatography (UHPLC) system (Duisburg, Germany) and parameter settings for the detection of AAT peptides at a Triple Quad 5,500+ mass spectrometer (AB SCIEX, Framingham, MA, USA) were adopted from previous publications (19, 24).

LC-MS/MS data processing was done with Analyst Software (version 1.6.2 and 1.7.1). Peak integration was reviewed individually and if applicable analyte peak areas were normalized to the peak area of their respective internal standards. Concentrations were calculated from a quadratic fit standard curve with 1/x*x weighting. All analytes with their corresponding quantification range are as previously published (17).

### 2.5 Statistics

Statistical analyses were conducted using R software (version 4.4.2), and data visualization was performed with Prism (version 9.1.2, GraphPad Software). Quantitative variables were summarized as mean [standard deviation (SD)], median [interquartile range (IQR)], and minimum–maximum values. Qualitative variables were described using absolute numbers and percentages. For laboratory measurements below the lower limit of quantification, values were imputed by dividing the quantification limit by the square root of two. Logarithmic transformation was applied to variables with skewed distributions where appropriate. Given the exploratory nature of this study, which aimed to identify potential biomarker candidates rather than to test predefined hypotheses, we did not apply formal correction for multiple comparisons. Considering the limited sample size, strict adjustments would increase the risk of type II errors; therefore, unadjusted *p*-values are reported, and all associations are interpreted with appropriate caution. Spearman's rank correlation coefficient was used to assess associations between quantitative variables, and corresponding 95% confidence intervals were reported. Comparisons between quantitative and categorical variables were performed using the Mann–Whitney *U*-test. For comparisons between two binary variables, the Chi-square test and Cramér's V were applied, with 95% confidence intervals provided. Due to the exploratory character of this study, no correction for multiple comparisons was performed. A *p*-value < 0.05 was considered statistically significant.

## 3 Results

### 3.1 Quantification of total AAT and circulating AAT-derived peptides in plasma

**Figure 1:**
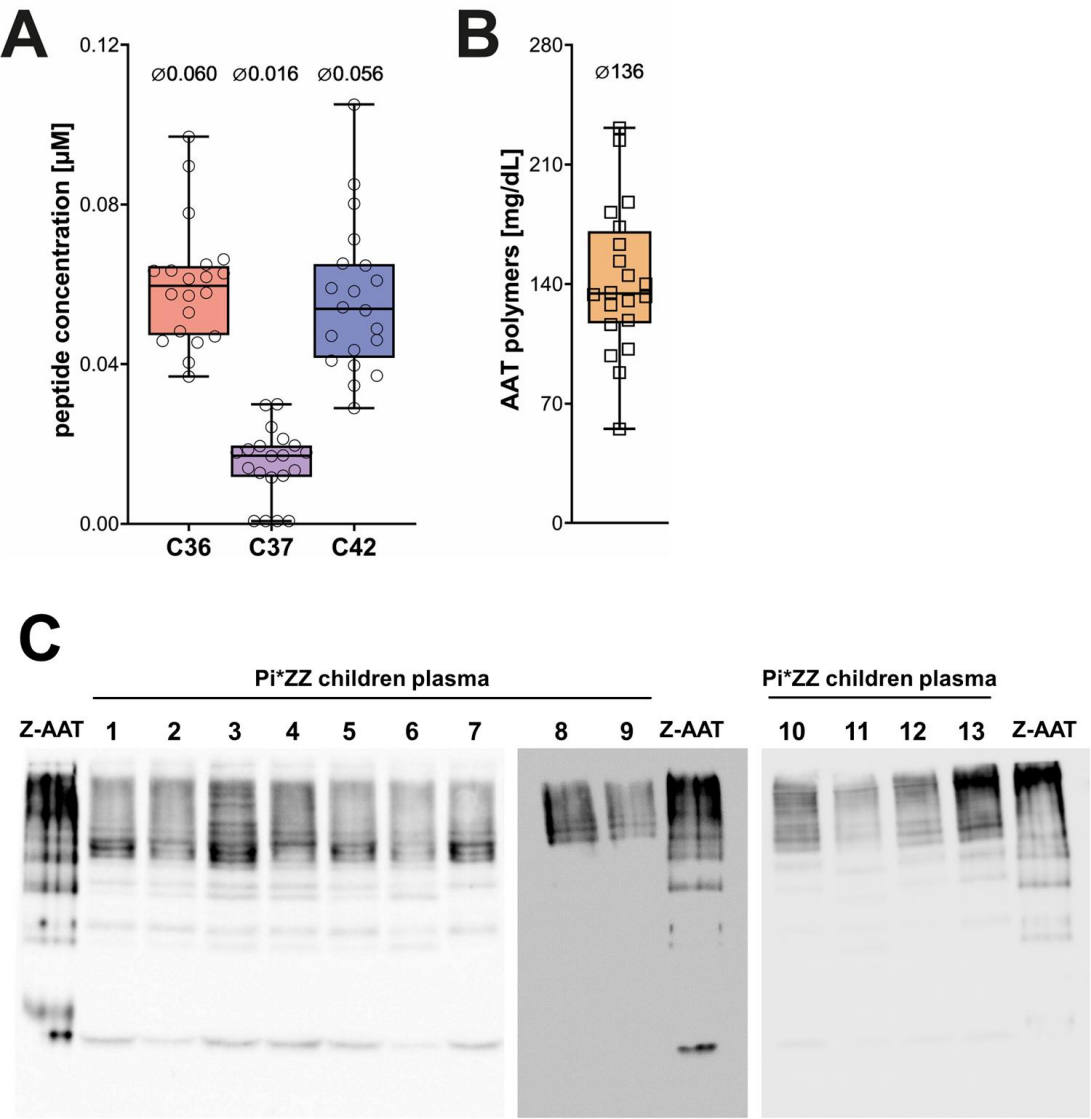
Plasma level of C-terminal peptides and polymeric AAT in pediatric samples. EDTA-plasma was analyzed by LC-MS/MS for AAT peptide analysis or by Western Blot for polymeric AAT. **(A)** The three peptides C36, C37, and C42 could be consistently found at micromolar concentrations in all 20 samples of pediatric Pi*ZZ carrier. Peptide level below the methods lower quantification limit (LLOQ = 0.01 µM for C37 or 0.025 µM for C36 and C42) were imputed by dividing LLOQ by the square root of 2 (*n* = 4 for C37). **(B)** Representation of the concentration (mg/dL) of AAT polymers among the 20 pediatric AATD patients. Boxplots represent the median and interquartile range with whiskers indicating the lowest and highest values. The median concentration (in µM for AAT peptide and mg/dL for AAT polymers) is indicated above each individual analyte. **(C)** Plasma samples were separated on native 7.5% polyacrylamide gels, blotted onto PVDF membranes and polymeric AAT was visualized using the LG96 monoclonal mouse antibody. Z-AAT purified from human plasma (1.5 µg/mL) was loaded onto each gel as an internal standard for semiquantitative densitometric analysis. Representative Western Blot images are shown. AAT, alpha-1 antitrypsin.

In the pediatric plasma samples only three C-terminal AAT peptides, C36, C37 and C42, cloud be consistently quantified (Figure 1). Other peptides were not detected (C22 and C39) or below the lower limit of quantification (C40, C43, C44 and C45). As for our previous analysis in adult patients with AATD (22), we investigated whether the total AAT concentration was related to the levels of measured AAT peptides (C36, C37, C42). Because most total AAT levels were at or below the linear range of the assay, patients were stratified using a median total AAT threshold of 0.3 g/L. No significant differences in peptide concentrations were detected between patients with total AAT levels ≥0.3 g/L and those with levels <0.3 g/L (Table 2). Furthermore, we investigated whether the patients' age or sex influenced the circulating AAT protein level. Patients with AAT levels at or above 0.3 g/L tent to be younger than those with levels below this threshold [median [IQR], 7.25 [7] years *vs.* 10.21 [3.54]], however, the difference was not statistically significant (Mann–Whitney-*U*-Test). Furthermore, there was no significant association with sex in this cohort [Cramérs V = 0.19, (0.02, 0.6), *p* = ns].

**Table 2:** Total AAT (categorized) and AAT peptides.

Total AAT	log(C36) [μM]		log(C37) [μM]		log(C42) [μM]	
	Median (IQR)	*p*-value^a^	Median (IQR)	*p*-value^a^	Median (IQR)	*p*-value^a^
≥0.3 g/L	−2.78 (0.18)		−4.02 (0.38)		−2.84 (0.29)	
<0.3 g/L	−2.98 (0.37)	ns	−4.32 (0.75)	ns	−3.18 (0.37)	ns

AAT, alpha-1-antitrypsin; C, C-terminal alpha-1 antitrypsin peptides; IQR, interquartile range; ns, not significant.

a P-value for Mann–Whitney-*U*-Test comparing total AAT (categorized) and AAT peptides.

### 3.2 Association between circulating plasma AAT polymers and C-terminal AAT peptides

**Figure 2:**
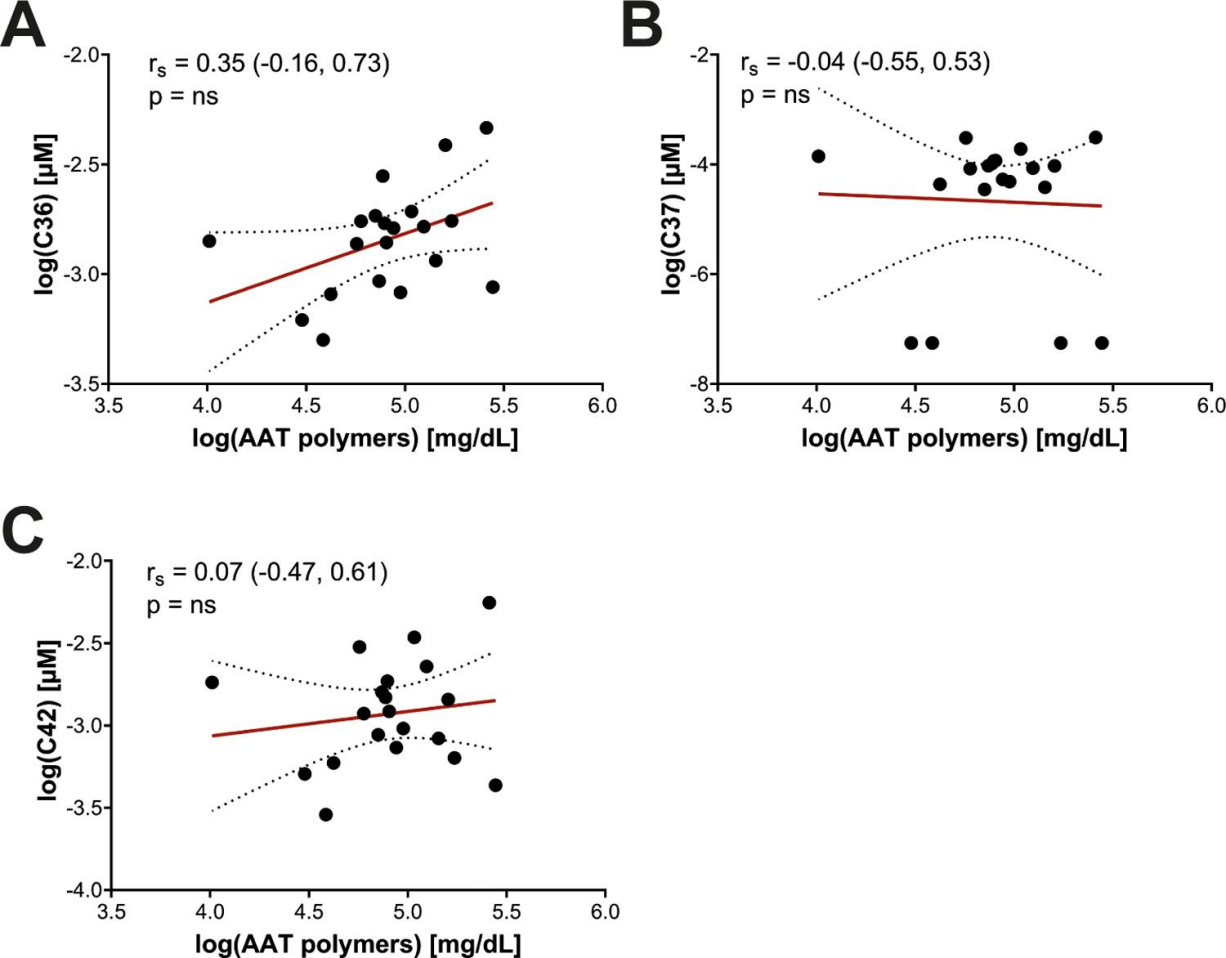
Association between AAT peptides and circulating AAT polymers in pediatric Pi*ZZ carriers. The concentration of AAT peptides (in µM) and polymers (in mg/dL) were log transformed before further analysis. Values failed Shapiro–Wilk normality test and correlations were calculated using Spearmen's test; the correlation factor r_s_ with the 95%-confidence interval and *p*-value between AAT peptides and polymers are given. AAT, alpha-1 antitrypsin; ns, not significant.

Semi-quantitative measurement of circulating Z-AAT polymers was performed using a native PAGE/Western blot method with the monoclonal antibody LG9, as previously described (25). Densitometric signals of Z-AAT polymers from patient samples were normalized to an internal polymer standard included on each blot, ensuring comparable semi-quantification across experiments (Figure 1C). Because C-terminal AAT peptides may arise from proteolytic cleavage of misfolded or aggregated AAT species, including polymers, we investigated whether plasma polymer levels correlated with circulating peptide concentrations. However, no significant associations were observed between Z-AAT polymer levels and any of the measured C-terminal AAT peptides (Figure 2). These findings suggest that circulating peptide generation is unlikely to be directly driven by polymer burden *in vivo* and may instead reflect independent proteolytic processes or distinct disease-related mechanisms.

### 3.3 Levels of AAT polymers and C-terminal peptides in relation to laboratory parameters of the liver

**Figure 3:**
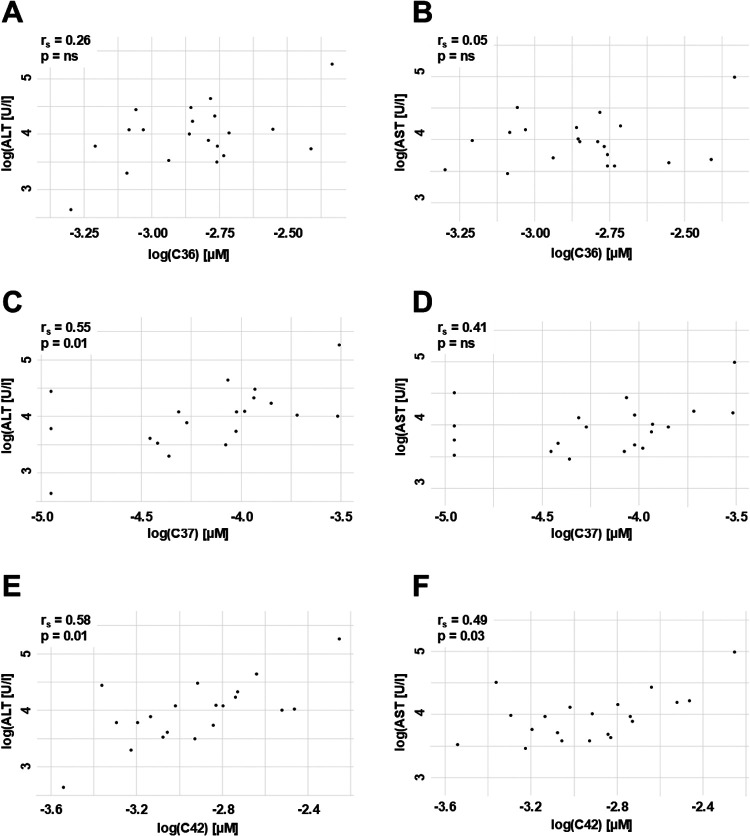
Correlation of AAT peptides to ALT and AST. The concentration of AAT peptides (in µM) and ALT and AST (in U/I) were log transformed for the analysis. Values failed Shapiro–Wilk normality test and correlations were calculated using Spearmen's test. The correlation factor r_s_ with the 95%-confidence interval and *p*-value between AAT peptides and ALT or AST are given. AAT, alpha-1 antitrypsin; ALT, alanine aminotransferase; AST, aspartate aminotransferase; ns, not significant.

Since AAT peptides were independent from the Z-AAT protein concentration, we analyzed the relationships between circulating AAT polymers and C-terminal AAT peptides to laboratory parameters including ALT (alanine aminotransferase), AST (aspartate aminotransferase), GGT (*γ*-glutamyltransferase), total bile acids, and platelet counts (Table 3). C36 showed no significant associations with any of the analyzed parameters. Both C37 [r_s_ = 0.55; 95% CI (0.1, 0.82)] and C42 [r_s_ = 0.58; 95% CI (0.11, 0.85)] were positively correlated with ALT, while C42 also showed a positive association with AST [r_s_ = 0.49; 95% CI (−0.05, 0.87)] (Figure 3).

**Table 3:** AAT polymers/AAT peptides and Age, ALT, AST, GGT, bile acid and thrombocytes.

Variable	Log (AAT polymers) [mg/dL]		Log (C36) [μM]		Log (C37) [μM]		Log (C42) [μM]	
	r_s_^a^	*p*-value^b^	r_s_^a^	*p*-value^b^	r_s_^a^	*p*-value^b^	r_s_^a^	*p*-value^b^
Age (years)	0.22 (−0.35, 0.66)	ns	−0.18 (−0.6, 0.36)	ns	−0.74 (−0.93, −0.34)	<0.001	−0.79 (−0.92, −0.43)	<0.001
Log (ALT) [U/L]	0.40 (−0.07, 0.77)	ns	0.26 (−0.27, 0.68)	ns	0.55 (0.1, 0.82)	0.01	0.58 (0.11, 0.85)	0.01
Log (AST) [U/L]	0.44 (−0.05, 0.81)	ns	0.05 (−0.56, 0.56)	ns	0.41 (−0.09, 0.79)	ns	0.49 (−0.05, 0.87)	0.03
Log (GGT) [U/L]	0.49 (0.05, 0.78)	0.03	0.32 (−0.11, 0.68)	ns	0.08 (−0.4, 0.51)	ns	0.25 (−0.27, 0.64)	ns
Log (Bile acid) [μmol/L]	0.32 (−0.37, 0.77)	ns	0.12 (−0.53, 0.74)	ns	−0.20 (−0.72, 0.47)	ns	−0.18 (−0.77, 0.53)	ns
Thrombocytes [G/L]	0.28 (−0.16, 0.64)	ns	0.01 (−0.44, 0.46)	ns	0.10 (−0.4, 0.57)	ns	0.11 (−0.38, 0.56)	ns

AAT, alpha-1-antitrypsin; ALT, alanine aminotransferase; AST, aspartate aminotransferase; GGT, γ-glutamyltransferase; ns, not significant.

a Spearman's rho with 95%-confidence interval.

b *P*-value for spearman correlation between AAT polymers/AAT peptides and age, ALT, AST, GGT, bile acid and thrombocytes.

Circulating AAT polymers were positively associated with GGT [rs = 0.49; 95% CI (0.05, 0.78)]. No significant correlations were observed between AAT polymers or any of the analyzed peptides and bile acid levels or platelet counts (Table 3).

### 3.4 AAT polymers and peptides in relation to clinical characteristics of the patients

We next examined associations between circulating AAT polymers, detectable AAT-derived peptides, and patient characteristics, including sex, age, liver disease severity, UDCA therapy, and history of *icterus prolongatus* (Table 4). AAT polymer levels and peptide concentrations did not differ significantly with respect to sex, disease severity, or *icterus prolongatus*. However, the peptides C37 [r_s_ = −0.74; 95% CI (−0.93, −0.34)] and C42 [r_s_ = −0.79; 95% CI (−0.92, −0.43)] were negatively associated with age, suggesting that their levels decrease independently from the total AAT level as children with AATD grow older. On the other hand, the peptide C36 did not show a correlation to the patients’ age. In addition, only levels of the peptides C37 (*p* = 0.02) and C42 (*p* = 0.004) were significantly lower in children receiving UDCA therapy compared with untreated patients. However, no significant association was observed between advanced disease severity (grades 3–4) and UDCA treatment (Cramér's V = 0.26, 95% CI: 0.01–0.68, *p* = ns).

**Table 4:** AAT polymers/AAT peptides and sex, severity, UDCA therapy and icterus prolongatus.

Variable	Log (AAT polymers) [mg/dL]		Log (C36) [μM]		Log (C37) [μM]		Log (C42) [μM]	
	Median (IQR)	*p*-value^a^	Median (IQR)	*p*-value^a^	Median (IQR)	*p*-value^a^	Median (IQR)	*p*-value^a^
Sex								
Female	4.94 (0.2)		−2.78 (0.1)		−4.07 (0.34)		−2.91 (0.49)	
Male	4.85 (0.4)	ns	−2.94 (0.44)	ns	−4.31 (0.43)	ns	−3.02 (0.34)	ns
Severity								
3	4.91 (0.24)		−2.85 (0.26)		−4.07 (0.48)		−2.91 (0.34)	
4	4.78 (0.27)	ns	−2.76 (0.24)	ns	−4.08 (0.78)	ns	−2.93 (0.53)	ns
UDCA therapy								
No	4.89 (0.36)		−2.78 (0.21)		−3.89 (0.37)		−2.69 (0.26)	
Yes	4.9 (0.28)	ns	−2.9 (0.31)	ns	−4.34 (0.52)	0.02	−3.07 (0.28)	0.004
Icterus prolongatus								
No	4.9 (0.33)		−2.86 (0.29)		−4.19 (0.52)		−2.97 (0.4)	
Yes	4.89 (0.29)	ns	−2.76 (0.18)	ns	−4.05 (0.44)	ns	−2.89 (0.37)	ns

AAT, alpha-1-antitrypsin; IQR, interquartile range; ns, not significant; UDCA, ursodeoxycholic acid.

a *P*-value for Mann–Whitney-*U*-Test comparing AAT polymers/AAT peptides and sex, severity, UDCA therapy and Icterus prolongatus.

## 4 Discussion

In this study, we examined circulating AAT polymers and C-terminal AAT-derived peptides in 20 children with Pi*ZZ AATD (median age 7.2 years, range 0.5–14.4). Unlike in our previous report in untreated adults with AATD-related lung disease, we consistently detected three distinct C-terminal peptides (C36, C37, and C42) in the plasma of our pediatric cohort, all of whom presented with clinically stable liver disease of mild to moderate severity (22). Although routine laboratory assays limited precise quantification of AAT concentrations below 0.3 g/L, total AAT levels in these children were comparable to those previously observed in adult Pi*ZZ patients. Notably, in contrast to adults, no association was found between circulating peptide levels and total AAT concentration in children with Pi*ZZ, although one cannot exclude that the dichotomous categorization of AAT levels (< 0.3 g/L vs. ≥ 0.3 g/L) may have reduced the sensitivity to detect more subtle associations.

The relationship between circulating peptide levels and total AAT concentration may vary depending on age, the underlying condition, and other factors. For example, in adults with COPD, circulating AAT peptides were detectable in Pi*MM but absent or below the limit of quantification in Pi*ZZ individuals. After AAT augmentation therapy in Pi*ZZ carriers, peptide levels increased above the quantification limit, reflecting their dependence on total AAT concentration. Similarly, in patients with non-small cell lung cancer and normal AAT protein levels, individual peptides showed varying correlations with AAT concentration; notably, C42 and C37, both products of MMP cleavage, exhibited stronger correlations (26). These findings indicate that although AAT peptides are derived from AAT protein, their formation is additionally influenced by disease-specific proteases and other contextual factors.

In contrast, we observed a negative association between peptide concentrations and age in our pediatric cohort, a pattern not seen in adults with Pi*ZZ-related lung disease (22). Although total AAT levels in children showed a trend toward age-related decline, this did not reach statistical significance. These observations suggest that AAT-derived peptides may follow an independent, age-related distribution pattern in AATD. Notably, the natural course of Pi*ZZ AATD-related liver disease exhibits a bimodal distribution, with pediatric liver biopsies often demonstrating higher fibrosis grades than adult specimens (27). Our findings thus highlight these age- and organ-specific differences in AATD pathophysiology and indicate that C-terminal AAT-derived peptides may serve as promising candidate markers for monitoring liver status in children, potentially providing complementary information to conventional laboratory measures of total AAT. Importantly, peptides C37 and C42 were significantly associated with liver transaminases, with C37 correlating with ALT and C42 with both ALT and AST. Because elevated transaminases reflect hepatocellular injury, these results indicate that circulating AAT-derived peptides may reflect the extent of liver damage. Mechanistically, this may involve enhanced proteolytic processing of AAT during hepatocellular stress, leading to the release of distinct C-terminal fragments.

Clinically, such peptides may therefore serve not only as indicators of injury severity but also as dynamic markers reflecting the pathogenic processes underlying pediatric AATD-related liver disease. Supporting this concept, we observed a negative association between peptide levels and the use of UDCA. While UDCA is commonly prescribed in cholestatic liver diseases, including AATD (23, 28), its use in pediatric AATD remains off-label and lacks definitive evidence of benefit. Nonetheless, clinical improvement in transaminase levels is often reported during UDCA treatment, and potential benefits in AATD have been suggested in previous studies (29, 30). Thus, the lower levels of C37 and C42 in UDCA-treated patients may reflect reduced hepatocellular injury. By shifting the bile acid pool toward more hydrophilic and less toxic species, UDCA likely diminishes hepatocyte and cholangiocyte membrane damage, which in turn may reduce the leakage of intracellular proteases and lower extracellular protease activity, ultimately decreasing the generation of circulating C-terminal AAT-derived peptides (31).

The absence of an association between peptide concentrations and liver disease severity in our cohort may reflect the small sample size and limitations of our severity classification. Additionally, this pilot study did not include patients with advanced liver disease. Building on previous findings that C-terminal AAT peptides increase under inflammatory conditions, it is plausible that peptide levels may be further elevated in pediatric patients with more advanced liver disease (17). Accordingly, a prospective study monitoring circulating AAT peptides in children and adolescences with Pi*ZZ could help confirm the relationships between peptide formation and clinical progression of liver disease.

Generation of C-terminal AAT peptides, such as C37 and C42, depends on specific matrix metalloproteinases (MMPs), which contribute to liver fibrosis; for example, MMP-3 is altered in children with AATD, and MMP-7 is upregulated in pediatric biliary atresia (32). Leung et al. reported elevated MMP-7 levels in AATD patients with increased liver stiffness, linking MMP activity to disease progression. Hence, peptides C37 and C42 may reflect dysregulated extracellular matrix remodeling driven by enhanced MMPs activity. Notably, bile acid therapy has been shown in cellular and rodent models to reduce hepatic MMP activity and thereby preserve hepatocyte function. This mechanism may account for the lower circulating levels of C37 and C42 observed in our pediatric patients receiving UDCA (27, 33). It is noteworthy that in our cohort, the serine protease-derived peptide C36 showed no association with liver transaminases or UDCA therapy. This contrasts with previous studies linking C36 primarily to systemic inflammation and neutrophil function, suggesting that its generation may reflect extrahepatic processes rather than hepatocellular injury (17, 19, 34). Pediatric liver disease in AATD is generally characterized by neutrophil-independent histopathological features such as bile duct proliferation, steatosis, and portal fibrosis (35). Whether AAT peptides (C36, C37, C40, C42) are detectable in children without AATD, and whether the observed age-related decline in peptide levels is specific to AATD, remains unknown. Addressing this question is critical to establishing their potential utility as predictors of disease severity in pediatric AATD.

As previously reported (13), we detected circulating AAT polymers in all Pi*ZZ patients, well-established contributors to hepatocellular stress and liver pathology in AATD. In Pi*ZZ AATD adults, plasma polymer levels have been shown to correlate with liver stiffness (11, 12). More recently, Teckman et al. demonstrated an association between circulating polymer concentrations and liver disease severity in a large pediatric AATD cohort (15). Similarly, as observed for C-terminal AAT peptides, polymer levels showed no association with clinical disease severity in our cohort. It is important to note, however, that Teckman et al. reported significant associations mainly in patients with clinically evident portal hypertension (CEPH), whereas our cohort included only children without CEPH (15). This distinction is relevant, as the majority of these latter Pi*ZZ children fall into the category of milder disease expression and early identification of those at risk remains a critical unmet need (6).

Nonetheless, we identified a positive association between GGT levels and circulating AAT polymers, a relationship that has also been reported in adult Pi*ZZ patients (12, 36). Furthermore, Teckman et al. demonstrated that elevated GGT and polymer levels during infancy are predictive of later CEPH development (15). However, the extent and robustness of the correlation between GGT and polymers in that study were not fully elaborated. Collectively, these findings suggest that AAT polymer levels may hold additional prognostic value in Pi*ZZ patients without CEPH. Larger prospective studies in Pi*ZZ children without CEPH are needed to validate our findings.

Several limitations should be acknowledged, particularly the exploratory nature of this study, the relatively small cohort size, and the lack of prospective follow-up to assess disease progression. In Germany, it is estimated that approximately 400 newborns per year carry the Pi*ZZ genotype (37). However, the clinical presentation during childhood is highly variable, and only about one in ten Pi*ZZ infants develops clinically apparent liver disease in the neonatal period or early childhood. Consequently, diagnosis is often delayed or may not occur at all during childhood, which substantially limits the availability of well-characterized pediatric patients for observational studies.

Future studies with larger cohorts, including age-matched control groups of wild-type (Pi*MM) children and individuals with other AATD genotypes, such as Pi*SZ, which typically cause less liver damage are necessary to confirm these initial observations. Such studies would enable a more comprehensive evaluation of the predictive potential of AAT peptides and the role of MMP activity in pediatric AATD-related liver disease. Nonetheless, our study represents the first robust, hypothesis-generating analysis in a clinically stable pediatric cohort, capturing subtle disease processes and supporting the broader relevance of these findings.

## Data Availability

The original contributions presented in the study are included in the article/Supplementary Material, further inquiries can be directed to the corresponding author.
