# Supplementary material for: Circulating C-terminal peptides and polymers of alpha-1 antitrypsin as putative markers of pediatric Pi*ZZ liver disease
Source: Front Pediatr. 2025 Dec 10;13:1717317. doi: 10.3389/fped.2025.1717317 (PMC12728065; doi:10.3389/fped.2025.1717317)
Supplement: Supplementary file 1 [file Table1.docx]

**Supplementary Tables**

**Supplementary Table S1.** Definition of liver disease groups

| group of liver disease | Criteria |
| --- | --- |
| 1: liver transplanted^†^ | - liver transplantation due to AATD |
| 2: severe liver disease^†^ | - persistent^1^ portal hypertension (PHT)² - liver failure - cirrhosis in biopsy or sonography |
| 3: moderate liver disease* | - persistent^1^ abnormal liver enzymes (ALT or GGT ≥2N³) - persistent^1^ pathological signs in sonography (hepatomegaly/ splenomegaly/ liver parenchymal remodelling) |
| 4: mild or no liver disease^†^ | - no persistent^1^ PHT² or cirrhosis of the liver - normal or almost normal liver biology in sonography - no persistent pathologies and laboratory results with mildly abnormal or normal liver enzymes (ALT and GGT < 2N³) |

AATD, alpha-1-antitrypsin deficiency; PHT, portal hypertension; ALT, alanine aminotransferase; GGT, Gamma-glutamyl transferase

**^†^** Group 1-3: ≥1 criteria had to be fulfilled, group 4: includes all patients who do not meet criteria of group 1-3 and thus all criteria from group 4 had to be fulfilled) ^1^ persistent defined as at least on the last two check-up occasions before evaluation and with an interval of maximum 2 years between the two measurements ² PHT evidenced by persistent platelets below 150 G/l, pathological coagulation (change in Quick/INR/Thrombin time, prothrombin time or factor V), oesophageal varices, ultrasound/doppler ultrasound with portosystemic derivations or reverse portal flow ³ More/less than twice the upper limit of normal
